# Protocol digest of a randomized controlled trial comparing transverse colectomy with extended right hemicolectomy for Stage II/III transverse colon cancer: JCOG2304 (TACTICS)

**DOI:** 10.1093/jjco/hyag084

**Published:** 2026-05-21

**Authors:** Tetsuya Sekita, Tomonori Akagi, Ryo Sadachi, Takumi Hasegawa, Hidefumi Shiroshita, Haruhiko Fukuda, Takeshi Suto, Akio Shiomi, Manabu Shiozawa, Shunsuke Tsukamoto, Yusuke Sano, Ryosuke Kita, Yukihide Kanemitsu, Masafumi Inomata, Japan Clinical Oncology Group Colorectal Cancer Study Group

**Affiliations:** Japan Clinical Oncology Group Data Center/Operations Office, National Cancer Center Hospital, Tokyo 104-0045, Japan; Department of Gastroenterological and Pediatric Surgery, Oita University Faculty of Medicine, Oita 879-5593, Japan; Japan Clinical Oncology Group Data Center/Operations Office, National Cancer Center Hospital, Tokyo 104-0045, Japan; Department of Gastroenterological and Pediatric Surgery, Oita University Faculty of Medicine, Oita 879-5593, Japan; Department of Gastroenterological and Pediatric Surgery, Oita University Faculty of Medicine, Oita 879-5593, Japan; Japan Clinical Oncology Group Data Center/Operations Office, National Cancer Center Hospital, Tokyo 104-0045, Japan; Department of Gastroenterological Surgery, Yamagata Prefectural Central Hospital, Yamagata, 990-2292, Japan; Division of Colon and Rectal Surgery, Shizuoka Cancer Center, Shizuoka, 411-8777, Japan; Department of Colorectal Surgery, Kanagawa Cancer Center, Yokohama, 241-8515, Japan; Department of Colorectal Surgery, National Cancer Center Hospital, Tokyo 104-0045, Japan; Japan Clinical Oncology Group Data Center/Operations Office, National Cancer Center Hospital, Tokyo 104-0045, Japan; Japan Clinical Oncology Group Data Center/Operations Office, National Cancer Center Hospital, Tokyo 104-0045, Japan; Department of Colorectal Surgery, National Cancer Center Hospital, Tokyo 104-0045, Japan; Department of Gastroenterological and Pediatric Surgery, Oita University Faculty of Medicine, Oita 879-5593, Japan

**Keywords:** transverse colectomy, extended right hemicolectomy, Stage II/III transverse colon cancer, relapse-free survival, quality of life

## Abstract

Transverse colon cancer is traditionally treated with extended right hemicolectomy (EHC), which involves division of the middle colic, right colic, and ileocolic arteries, along with removal of the terminal ileum and right colon. Transverse colectomy (TC) is a limited resection removing only 10 cm of bowel on either side of the tumor, along with resection of the middle colic artery and regional lymphadenectomy. This approach may contribute to the preservation of bowel function without compromising oncologic adequacy. This multicenter, randomized, phase III trial is designed to evaluate the non-inferiority of TC compared with EHC in terms of relapse-free survival among patients with Stage II/III transverse colon cancer whose main feeding artery is the middle colic artery. In total, 510 patients will be enrolled across major Japanese centers over 5 years. The trial has been registered with the Japan Registry of Clinical Trials (jRCT1030240479).

## Teaser

This randomized phase III trial will confirm non-inferiority of transverse colectomy versus extended right hemicolectomy for relapse-free survival of Stage II/III transverse colon cancer supplied by the middle colic artery.

## Introduction

In Japan, colorectal cancer (CRC) is a major cause of cancer incidence and mortality. In 2014, CRC accounted for ~134 000 new cases, and in 2017, it caused ~50 000 deaths, making it the second leading cause of cancer-related deaths overall, and the leading cause in women [1]. As well, CRC ranks globally as the second leading cause of cancer-related deaths. In the United States, over 150 000 new cases are diagnosed annually, with ˃50 000 deaths [2]. In Europe, CRC is similarly a top cause of cancer mortality, emphasizing the worldwide need for continued research and more effective treatments [3]. In Japan, colon cancer constitutes a substantial proportion of CRC. Population-based data from the Surveillance, Epidemiology, and End Results (SEER) program have shown that the 5-year overall survival rate for colon cancer is ~90% for localized disease, 70% for regional disease, and 15% for distant disease [4]. These data underscore the need for further therapeutic development to treat advanced disease stages.

Endoscopic resection is commonly employed for Stage 0 and some Stage I (minimally invasive) CRCs. Surgical resection is the standard of care for Stage II to Stage III disease with D3 lymphadenectomy, involving identification of the feeding vessels and corresponding lymphadenectomy [5]. For transverse colon cancers predominantly supplied by the middle colic artery, the standard procedure both domestically and internationally has been extended right hemicolectomy (EHC), which includes dissection and resection along the ileocolic, right colic, and middle colic arteries, resulting in a relatively extensive resection. EHC involves ileocolic anastomosis, which has been considered advantageous over colocolic anastomosis from the standpoint of postoperative blood supply. However, recent advances in surgical technology, such as stapling devices and intraoperative fluorescence imaging using indocyanine green, have enabled reliable colocolic anastomosis [6]. Several anatomical and clinical studies have demonstrated that the lymphatic drainage of colon cancer generally follows the distribution of feeding arteries. In transverse colon cancer primarily supplied by the middle colic artery, lymph node metastasis is predominantly observed within the middle colic territory, whereas metastasis to the ileocolic and right colic regions appears to be relatively infrequent [7, 8]. These findings suggest that extensive lymphadenectomy involving the ileocolic and right colic territories may not always be necessary and support the oncological adequacy of a more limited resection focusing on the middle colic artery territory.

Consequently, an increasing number of institutions have adopted transverse colectomy (TC), a function-preserving surgery that preserves the ileocecal region and shortens the resected bowel length by transecting only the middle colic artery. As a result, variability in surgical approaches to transverse colon cancer has emerged both in Japan and abroad.

There are reports suggesting that TC is not inferior to EHC in terms of oncological outcomes [9–12]. However, with the exception of the analysis from the U.S. National Clinical Database [12], most of these findings come from small case studies. Additionally, a subgroup analysis of the Japan Clinical Oncology Group (JCOG) JCOG1006 trial [13], a randomized controlled trial evaluating appropriate surgical techniques for colon cancer, showed a trend favoring TC in terms of 3-year overall survival (OS) (100% vs. 97.8%) and relapse-free survival (RFS) (94.0% vs. 84.8%), although these results were not adequately validated. Therefore, there is a clear rationale for conducting a randomized controlled trial to further assess the safety and efficacy of TC.

TC is considered a function-preserving procedure due to its potential to reduce bowel resection length and preserve the ileocecal valve. Several retrospective studies have suggested that TC is associated with improved postoperative bowel function, including reduced diarrhea, fewer nighttime bowel movements, and overall improved quality of life [14]. It has also been reported to alleviate watery stools and symptoms of irritable bowel syndrome [15]. Although these studies were retrospective, their findings consistently suggest the benefit of functional preservation. Animal studies have indicated that resection of the ileocecal region, as performed in EHC, may lead to increased bile acid flow into the colon and disruption of the gut microbiota due to loss of the ileocecal valve [16]. Bile acids have been implicated as risk factors for colorectal carcinogenesis [17], and dysbiosis has been suggested to contribute to tumor initiation and progression [18].

Based on this background, this randomized phase III trial aims to confirm whether TC, a function-preserving and limited resection, is non-inferior to EHC, the current standard surgery, in terms of RFS, while also preserving postoperative bowel function in patients with Stage II/III transverse colon cancer primarily supplied by the middle colic artery. The significance of this study lies in prospectively evaluating the safety and efficacy of TC as a potential new standard procedure for Stage II/III transverse colon cancer.

The JCOG Protocol Review Committee approved this study protocol on 2 August 2024, and centralized ethical approval was obtained from the Institutional Review Board of the National Cancer Center on 19 September 2024. This trial was initiated in November 2024 and registered in the Japan Registry of Clinical Trials (jRCT) as jRCT1030240479 [https://jrct.mhlw.go.jp/latest-detail/jRCT1030240479].

## Protocol digest of JCOG2304

### Purpose

We will conduct a randomized phase III trial to confirm the non-inferiority of TC, a function-preserving limited resection, compared to EHC in terms of RFS for patients with Stage II/III advanced transverse colon cancer with the middle colic artery as the main feeding vessel.

### Study setting

**Figure 1:**
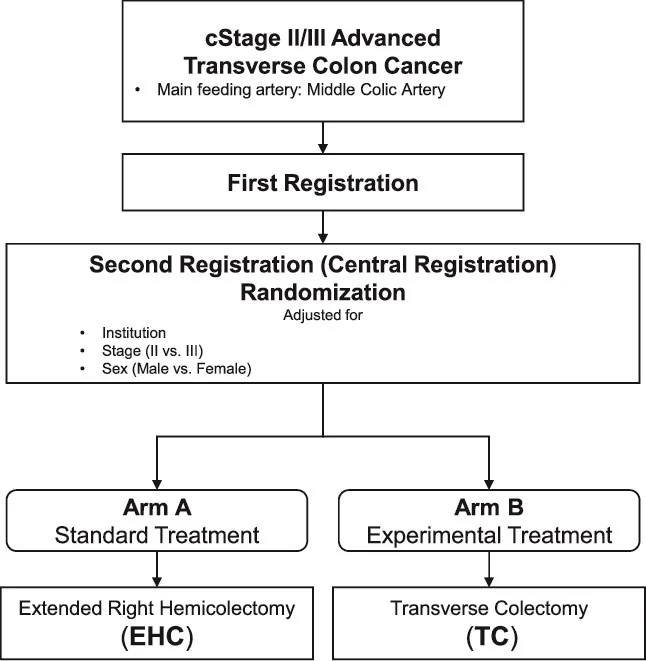
Study scheme.

A multi-institutional (62 specialized centers), open-label, randomized controlled phase III trial. The study scheme is shown in Fig. 1.

### Endpoints

The primary endpoint is RFS, defined as the time from randomization to the first event of either recurrence or death from any cause, whichever occurs first, and censored at the date of last contact for patients without an event. Secondary endpoints include OS, defined as the time from randomization to death from any cause; disease-free survival (DFS), defined as the time from randomization to the earliest occurrence of recurrence, second primary cancer, or death from any cause; and incidence of intraoperative and postoperative complications (classified as early [within 30 days after surgery] or late [after 31 days]).

Functional outcomes related to defecation and anorectal function are assessed in all treated patients at baseline (before treatment), and at 3 months, 6 months, 1 year, 3 years, and 5 years after registration. These include daytime and nighttime stool frequency, number of fecal incontinence episodes, abdominal bloating and pain assessed via Patient-Reported Outcomes version of the Common Terminology Criteria for Adverse Events (PRO-CTCAE) and CTCAE v5.0, and anorectal function scores assessed by the Wexner [19] and LARS [20] scoring systems.

### Eligibility criteria

First registration criteria:

(1) Histological diagnosis of endoscopic biopsy from a primary colorectal lesion as adenocarcinoma according to the 9th edition of the Japanese Classification of Colorectal Tumors (papillary adenocarcinoma, tubular adenocarcinoma, poorly differentiated adenocarcinoma, mucinous carcinoma, signet ring cell carcinoma, medullary carcinoma).
(2) Predominant location of the tumor in the transverse colon.
(3) Comprehensive diagnosis of clinical Stage II/III by contrast-enhanced computed tomography (CT).
(4) The tumor’s responsible blood vessel on contrast-enhanced CT (slice thickness of 1.25 mm or less), meeting both (i) and (ii):
  (i) Responsible blood vessel for the tumor: the middle colic artery.
  (ii) A single dominant vessel directly beneath the tumor, or a single dominant vessel within 10 cm distal to the tumor.
(5) No evidence of advanced organ invasion on contrast-enhanced CT.
(6) Absence of enlarged lymph nodes (short diameter 10 mm or more, short diameter 7 mm or more with irregular margins/internal heterogeneity/oval shape) outside the middle colic artery region (221, 222rt, 222lt, 223) on CT of the abdomen and pelvis with slice thickness of 1.25 mm or less.
(7) Comprehensive diagnosis by colonoscopy and imaging studies (barium enema, contrast-enhanced CT, or CT colonography), with no evidence of multiple carcinomas except for cTis and cT1a lesions (expected to remain in the submucosal layer and invade ˂1000 μm) considered curatively resectable by endoscopic resection.
(8) Aged 18–80 years old on the day of the registration.
(9) ECOG (Eastern Cooperative Oncology Group) performance status is 0 or 1.
(10) No history of intestinal resection.
(11) No intestinal obstruction requiring decompression treatment or perforation, except when the obstruction has been relieved by insertion of an ileus tube, transanal drainage tube, or stent.
(12) No history of chemotherapy for the underlying disease.
(13) No history of radiation therapy to the abdomen, including treatment for other types of cancer.
(14) Absence of familial adenomatous polyposis or Lynch syndrome based on the patient’s medical history.
(15) Latest blood examination result within 28 days prior to the first registration (the same weekday 4 weeks before registration is acceptable), meeting all of the following:
  (i) Neutrophils ≥1500/mm^3^.
  (ii) Platelets ≥100 000/mm^3^.
  (iii) Total bilirubin ≤2.0 mg/dL.
  (iv) Aspartate aminotransferase (AST) ≤ 100 U/L.
  (v) Alanine aminotransferase (ALT) ≤ 100 U/L.
  (vi) Serum creatinine ≤1.5 mg/dL.
  (vii) SpO_2_ ≥ 93% (room air)
(16) Written informed consent.

Second registration criteria:

(1) First registration for this trial and a second registration within 28 days of the first registration date (up to the same weekday 4 weeks later is acceptable).
(2) No liver metastasis or peritoneal dissemination on intra-abdominal observation.
(3) No other findings suggestive of lymph node metastasis outside the area of the middle colic artery.
(4) R0 resection is judged to be possible.

### Exclusion criteria

(1) Active multiple cancers (simultaneous multiple cancers and multiple cancers with a disease-free interval of 5 years or less, excluding Stage I prostate cancer, Stage 0, or Stage I laryngeal cancer that has had a complete response to radiotherapy, or cancer that has been completely resected with a 5-year relative survival rate equivalent to 95% or greater).
(2) Infectious diseases requiring systemic treatment.
(3) Fever of 38.0°C or higher at the time of registration.
(4) Pregnancy, possible pregnancy, within 28 days postpartum, or lactating. Males whose partners wish to become pregnant.
(5) Psychiatric disorders or psychiatric symptoms that interfere with daily life and make participation in the study difficult.
(6) Receiving continuous systemic administration (oral or intravenous) of steroids or other immunosuppressive drugs.
(7) Complicated with unstable angina (angina with onset or worsening within the last 3 weeks) or a history of myocardial infarction within the last 6 months.
(8) Poorly controlled valvular heart disease, dilated cardiomyopathy, or hypertrophic cardiomyopathy.
(9) Has one or more of the following complications diagnosed by chest CT: interstitial pneumonia, pulmonary fibrosis, or severe emphysema.

### Randomization

After the confirmation of eligibility criteria at the second registration, patients are randomized (1:1) to either Arm A (primary tumor resection by EHC) or Arm B (primary tumor resection by TC). Randomization is performed using the minimization method with a random component to balance the following factors: participating institution, disease Stage (II vs. III), and sex (male vs. female). The detailed procedure for randomization will not be disclosed to investigators at the participating institutions.

### Treatment methods

Patients are randomly assigned to either Arm A or Arm B at second registration, with protocol treatment initiated within 28 days of first registration. Second registration is performed intraoperatively via a web-based system after confirming eligibility criteria, and this marks the official start of protocol treatment. Surgical procedures may be performed through open, laparoscopic, or robot-assisted approaches. Assessment of anastomotic perfusion using indocyanine green fluorescence imaging is recommended but may be omitted if vascular perfusion can be clearly assessed by visual inspection or palpation. Any standard technique for bowel reconstruction, including hand-sewn or stapled anastomosis (e.g. overlap, functional end-to-end, or delta methods), and either intracorporeal or extracorporeal anastomosis, is acceptable. Postoperative adjuvant chemotherapy is not specified in this protocol, but its administration according to disease stage and in line with the 9th edition of the Japanese Classification of Colorectal Carcinoma is recommended. In Japan, oxaliplatin-based combination regimens are recommended for pathological Stage III disease, whereas fluoropyrimidine monotherapy is considered acceptable. Information on postoperative adjuvant chemotherapy, including regimen type and duration, will be collected as part of background clinical data. To ensure the allocated surgical procedure is appropriately performed, intraoperative photographs obtained after lymphadenectomy will be collected in both arms and centrally reviewed.

#### Arm A: extended right hemicolectomy

In Arm A, patients undergo EHC. Lymph node dissection is performed according to the 9th edition of the Japanese Classification of Colorectal Carcinoma, including dissection of the regional lymph nodes (Nos. 221, 222, and 223), as well as the additional nodal station Nos. 201, 202, 203, 211, 212, and 213. Vascular ligation involves division of the ileocolic, right colic (if present), and middle colic arteries. The oral resection margin for bowel resection is set at the terminal ileum, including resection of the ileocecal region, whereas the anal resection margin is placed 10 cm distal to the tumor.

#### Arm B: transverse colectomy

In Arm B, patients undergo TC. Lymph node dissection is performed in accordance with the same Japanese classification but is limited to the regional lymph node station Nos. 221, 222, and 223. Only the middle colic artery is ligated and divided. The oral bowel transection is made 10 cm proximal to the tumor, preserving the ileocecal region, and the anal transection is performed 10 cm distal to the tumor margin.

## Follow-up

Patients will undergo follow-up for at least 5 years after the completion of accrual. Assessments will be conducted every 3 months during the first 3 years, every 6 months for the subsequent 2 years, and annually after 5-year follow-up. Physical examinations and routine blood tests will be performed at each follow-up visit to evaluate general condition and organ function. Contrast-enhanced CT scans of the chest, abdomen, and pelvis will be performed every 6 months to assess for disease recurrence. Tumor markers (CEA and CA 19-9) will be monitored at the same intervals. Colonoscopy will be performed at years one, three, and five to detect local recurrence or secondary lesions. Late postoperative complications will be evaluated and graded using both CTCAE v5.0 and the Clavien-Dindo classification. Anorectal function and bowel symptoms, including defecation frequency and fecal incontinence, will be assessed using the Wexner score, LARS score, and PRO-CTCAE at predefined timepoints: 3 months, 6 months, 1 year, 3 years, and 5 years after surgery. After year five, annual outpatient visits will be recommended, and additional assessments will be performed as clinically indicated.

## Study design and statistical analysis

This randomized phase III trial aims to confirm the non-inferiority of TC, a limited resection, compared to EHC, the standard surgical procedure, in patients with Stage II/III advanced transverse colon cancer. The primary endpoint is RFS, which has been validated as a surrogate for OS in adjuvant CRC trials by Sargent *et al.* [21].

A 3-year RFS of 85% is assumed in the EHC arm, based on registry data from the Japanese Study Group for Postoperative Follow-up of CRC. A 3-year RFS of 86% is expected in the TC arm, based on subgroup analyses from the JCOG1006 trial [13], which showed no significant difference between EHC and TC (3-year RFS: 84.8% vs. 94.0%; 3-year OS: 97.8% vs. 100%), and a multicenter study by the Kanagawa Yokohama Colorectal Cancer group, in which TC was associated with better outcomes (3-year DFS: 81.2% vs. 60.0%).

In total, 510 patients (255 per arm) are required for this non-inferiority design. The required sample size was calculated using a Cox proportional hazards model within a non-inferiority framework, assuming a non-inferiority margin of 5% in 3-year RFS (corresponding to a hazard ratio [HR] of 1.37), a one-sided alpha of 5%, 70% power, a 5-year accrual period, and a 3-year follow-up. The non-inferiority margin of HR 1.37 was determined based on consensus within the study group and is considered clinically acceptable [22, 23]. A previous review of non-inferiority trials across various cancer types and interventions showed that a margin around HR 1.3 has been commonly adopted in studies using RFS or similar endpoints, even outside the context of adjuvant chemotherapy [24]. Therefore, the margin of HR 1.37 is considered reasonable and safe, given the expected risk difference.

The primary analysis for RFS will be conducted at 3 years of follow-up using a stratified Cox regression model, with stratification factors including clinical stage and sex. The stratified Cox proportional hazards model will be used to estimate the HR between the two arms. All statistical analyses will be conducted at the JCOG Data Center.

### Interim analysis and monitoring

One interim analysis is planned and will be conducted by the JCOG Data Center using data obtained from central monitoring after enrollment of approximately half of the planned number of patients. To control the overall one-sided type I error at 5%, multiplicity between the interim and final analyses will be adjusted using the Lan-DeMets α-spending function with the O’Brien and Fleming type boundary [25]. Early termination of the trial will be considered if the primary endpoint in the TC arm demonstrates statistical superiority over the EHC arm or if the interim results indicate that the continuation of the study is unlikely to demonstrate non-inferiority at the final analysis. Conditional power and predictive probability will be used as supportive tools in assessing futility [26, 27]. All interim results will be independently reviewed by the JCOG Data and Safety Monitoring Committee.

The JCOG Data Center will provide central monitoring to ensure study progress, data quality, and patient safety. In addition, the JCOG Audit Committee will conduct site visit audits as part of routine quality assurance across the study group. These audits will contribute to data integrity and compliance with protocol standards, although they are not specific to this trial.

## Data Availability

The data generated to support the findings of this study will be made available from the corresponding author upon reasonable request.

## Japan registry of clinical trials registration number

This trial has been registered at the jRCT as jRCT1030240479 [https://jrct.mhlw.go.jp/latest-detail/jRCT1030240479].

## Participating institutions in Japan (from North to South)

Tohoku University Hospital, Miyagi Cancer Center, Yamagata Prefectural Central Hospital, University of Tsukuba, Tochigi Cancer Center, Gunma Prefectural Cancer Center, National Defense Medical College, Saitama Cancer Center, Jichi Medical University Saitama Medical Center, Saitama Medical University International Medical Center, Saitama Medical University General Medical Center, National Cancer Center Hospital East, Chiba Cancer Center, National Cancer Center Hospital, Kyorin University Faculty of Medicine, Tokyo Medical University Hospital, Tokyo Metropolitan Cancer and Infectious Diseases Center Komagome Hospital, Tokyo Medical and Dental University, Jikei University Hospital, Cancer Institute Hospital of Japanese Foundation for Cancer Research, Toho University Ohashi Medical Center, Toho University Omori Medical Center, Nippon Medical School Hospital, Tokai University School of Medicine, Kanagawa Cancer Center, Kitasato University School of Medicine, Yokohama City University Medical Center, Niigata Cancer Center Hospital, Nagaoka Chuo General Hospital, Ishikawa Prefectural Central Hospital, Gifu University School of Medicine, Ogaki Municipal Hospital, Gifu Prefectural General Medical Center, Shizuoka Cancer Center, Aichi Cancer Center Hospital, Nagoya University School of Medicine, Fujita Health University, National Hospital Organization Kyoto Medical Center, Osaka University School of Medicine, Osaka International Cancer Institute, National Hospital Organization Osaka National Hospital, Osaka Medical and Pharmaceutical University, Toyonaka Municipal Hospital, Minoh City Hospital, Suita Municipal Hospital, Kansai Medical University Hospital, Kobe University School of Medicine, Kobe City Medical Center General Hospital, Kansai Rosai Hospital, Hyogo Medical University, Hyogo Cancer Center, Himeji Red Cross Hospital, Kurashiki Central Hospital, Hiroshima City Hiroshima Citizens Hospital, Hiroshima Prefectural Hospital, Hiroshima City Asa Citizens Hospital, Fukuyama City Hospital, National Hospital Organization Shikoku Cancer Center, Kochi Medical Center, Kurume University School of Medicine, Kumamoto University Hospital, Oita University Hospital.
